# The level of antiretroviral therapy (ART) adherence among orphan children and adolescents living with HIV/AIDS: A systematic review and meta-analysis

**DOI:** 10.1371/journal.pone.0295227

**Published:** 2024-02-21

**Authors:** Stanley Githaiga Kamau, Rita Akatusasira, Angella Namatovu, Emmanuel Kibet, Joseph Mayanja Ssekitto, Mohammed A. Mamun, Mark Mohan Kaggwa

**Affiliations:** 1 Faculty of Medicine, Mbarara University of Science and Technology, Mbarara, Uganda; 2 CHINTA Research Bangladesh, Savar, Dhaka, Bangladesh; 3 Department of Public Health and Informatics, Jahangirnagar University, Savar, Dhaka, Bangladesh; 4 Department of Public Health, University of South Asia, Dhaka, Bangladesh; 5 Department of Psychiatry and Behavioural Neurosciences, McMaster University, Hamilton, Canada; 6 Department of Psychiatry, Mbarara University of Science and Technology, Mbarara, Uganda; 7 Forensic Psychiatry Program, St Joseph’s Healthcare Hamilton, Hamilton, Ontario, Canada; Médecins Sans Frontières (MSF), SOUTH AFRICA

## Abstract

**Background:**

Many children and adolescents living with HIV have ended up as orphans. Due to HIV taking away their parents leaves them deprived of their most important social network and support, which predisposes them to poor adherence to antiretroviral therapy (ART). Various studies have shown poor adherence to ART among orphaned children and adolescents. This systematic review and meta-analysis, therefore, aims to determine the level of ART adherence among orphaned children and adolescents living with HIV/AIDS.

**Methods:**

This PROSPERO registered review (CRD42022352867) included studies from *PubMed*, *Google Scholar*, *Scopus*, *Web of Science*, *Africa Journal Online*, and selected HIV/AIDS journals from data inception to June 01, 2022. We included articles published in all languages that report the prevalence of adherence to ART among children and adolescent orphans (single parent orphans and/or double orphans) living with HIV/AIDS. We excluded qualitative studies, case studies, opinion papers, and letters to editors. We used the random-effect model to calculate the pooled prevalence of ART adherence based on the highest prevalence provided by the various methods in a particular study. We used the Joanna Briggs Institute Appraisal tool for the prevalence study to evaluate for risk of bias in the included studies. The Egger’s test was used to assess small study effects.

**Results:**

Out of 1087 publications identified from the various databases, six met the selection criteria. The included six studies had a total 2013 orphans living with HIV/AIDS. The pooled prevalence of ART adherence was 78∙0% (95% Confidence Interval: 67.4–87.7; *I*^2^ = 82.92%, *p*<0∙001) and ranged between 7∙6% and >95%, using one of the following methods: pill count, caregiver’s self-report, clinical attendance, and nevirapine plasma levels (above three μg/mL). The factors associated with adherence were pill burden, caregiver involvement, stunting, and caregiver relationship.

**Limitation:**

There was a high level of heterogeneity in the finding.

**Conclusion:**

Approximately four fifth of orphan children and adolescents living with HIV/AIDS adhere to ART. Strategies to improve adherence among this group should be prioritized, especially among the double orphaned children and adolescents.

## Introduction

The acquired immunodeficiency syndrome (AIDS) is a chronic condition caused by the human immunodeficiency virus (HIV), characterized by a damaged immune system and decreased immunity. The Joint United Nations Programme on HIV/AIDS estimated that in 2021, approximately 38.4 million (33.9 million–43.8 million) people worldwide were living with HIV/AIDS, with 1.5 million newly infected cases reported [1]. Among them, around 1.7 million (ranging from 1.3 to 2.1 million) were children aged 0–13 years [1]. In 2021, about 75% of HIV/AIDS cases received antiretroviral therapy (ART), with 52% of these cases being among children under 15 years old [1]. However, inadequate access to HIV prevention, care, and treatment services, as well as poor adherence to ART, continue to contribute to AIDS-related deaths, which averaged 0.65 million per day in 2021 [1, 2].

Adherence to ART is crucial for long-term survival, and it poses specific challenges among adolescents and children [3]. While children often exhibit better adherence due to their dependence on caregivers for healthcare, adolescents face unique struggles during their rapid psychosocial and physical transition, as they strive for independence and take on the responsibility of their own adherence [3]. The prevalence of ART adherence among children and adolescents varies significantly between countries. For example, a 2015 study done in Tanzania showed that the average ART adherence level was 70% among children and 84% among adolescents [4]. In 2018, assessing adherence by doses missed over the past week, 79% of the children had ART adherence in Uganda [5]. Another 2018 study in Zambia showed that ART adherence among adolescents was 71.8% [6]. Additionally, in a 2016 study in India, the adherence among children living with HIV/AIDS was 90.9% [7]. However, it is important to note that adherence rates also differ among different populations, such as adults in India, where a systematic review and meta-analysis reported an optimum ART adherence rate of 77% [8].

Achieving optimal adherence to ART is essential for better health outcomes and to prevent HIV transmission, especially among adolescents and children, in order to meet the UNAIDS targets of 95-95-95 by 2025 [9, 10]. Several barriers to ART adherence have been identified, including only the caregiver knowing the child`s serostatus, conflicts between the child and caregivers, orphanhood, male gender, divorced or widowed caregivers, and longer duration on ART [5, 11, 12]. Studies have shown variations in adherence rates based on orphanhood status, with higher non-adherence observed among orphans compared to children with both parents [13, 14]. For instance, double orphans had 41.7% adherence in a study conducted in Kigali, whereas 55.1% and 53.3% for maternal and paternal orphans, respectively [13]. Contextual factors such as psychological distance between caregivers and children and economic burden have been found to contribute to these variations [15]. Additionally, the role of caregivers in providing adherence support has been shown to differ based on orphanhood status, with paternal orphans receiving better support from biological mothers and maternal orphans receiving adequate support from grandmothers compared to double orphans [14]. A study on treatment outcomes among HIV-positive orphaned and non-orphaned children on ART revealed that orphans were more likely to have detectable viral loads due to lack of psychosocial support or stigma, which posed challenges for adherence [16]. Based on aforementioned discussion, orphanhood status may be a factor for long-term therapy response, even though a study in South Africa reported no effect of orphanhood status on ART adherence [17].

Despite the extensive literature on the effect of orphanhood on adherence, no review has synthesized the pooled prevalence of adherence to ART among children and adolescents. This systematic review and meta-analysis aims to determine the pooled prevalence of adherence to ART among children and adolescent orphans and summarize the factors associated with ART adherence from previously published literature. The findings will provide insights into the progress made towards achieving the UNAIDS target and enable comparisons with other vulnerable groups to inform adherence support and resource allocation. Following the guidelines from the Joanna Briggs Institute (JBI) for formulating a review question using the CoCoPop (Condition, Context, and Population) approach, we have developed the research question for this global systematic review and meta-analysis: “What is the level of adherence to ART among children and adolescent orphans (single parent orphans and/or double orphans) living with HIV/AIDS globally?”

## Methods

### Search strategy

The PRISMA (Preferred Reporting Items for Systematic Reviews and Meta-analyses) guideline was adhered to. This is a PROSPERO registered review (CRD42022352867) [18]. The Meta-analysis of Observational Studies in Epidemiology (MOOSE) guidelines for systematic reviews and meta-analysis of observational studies [19]. From May 30 –June 1, 2022, a systematic literature search was done in *PubMed*, *Scopus*, *Web of Science*, and *Africa Journal Online*. We also searched *Google Scholar* to retrieve preprints and articles not present in the previous databases. In addition, we performed a literature search in some of the HIV journals and online resources, that is, *AIDs and Behavior*, *The lancet HIV*, *African journal of AIDS research*, *AIDS research and therapy*, and also, searches of the relevant articles were done from the list of references of the initially retrieved papers. The following search terms were utilized: (i) Adherence OR Compliance, (ii) HIV OR AIDs, (iii) Orphans, and (iv) Children or adolescents OR teenagers OR children OR youth OR emancipated youth OR juveniles OR minors OR younger persons. The search strings from *PubMed* is presented in **S1 File** and its keywords were translated into other databases. To ensure inclusion of all related articles, the research team conduct another literature search from three databases (*PubMed*, *Embase*, *and Medline*).

### Study selection criteria

Screening was done by four authors (SGK, RA, AN, and EK) based on “Titles and Abstract” first. In pairs, the authors reviewed 50% of the records. Then, two independent (RA and SGK) reviewers evaluated the full-text article to confirm if the article was to be included or not. The articles included in this review, after adhering to the inclusion criteria, include (i) being a study about children and orphans living with HIV/AIDS, (ii) reporting level of adherence among orphan children and/or adolescents, and (iii) published in a peer-reviewed journal or preprint. We applied no language restrictions. We excluded case reports, case series, reviews, editorials, commentaries, and view papers. MMK settled any discrepancies at these stages following discussions with the team members.

### Data eligibility

**Fig 1:**
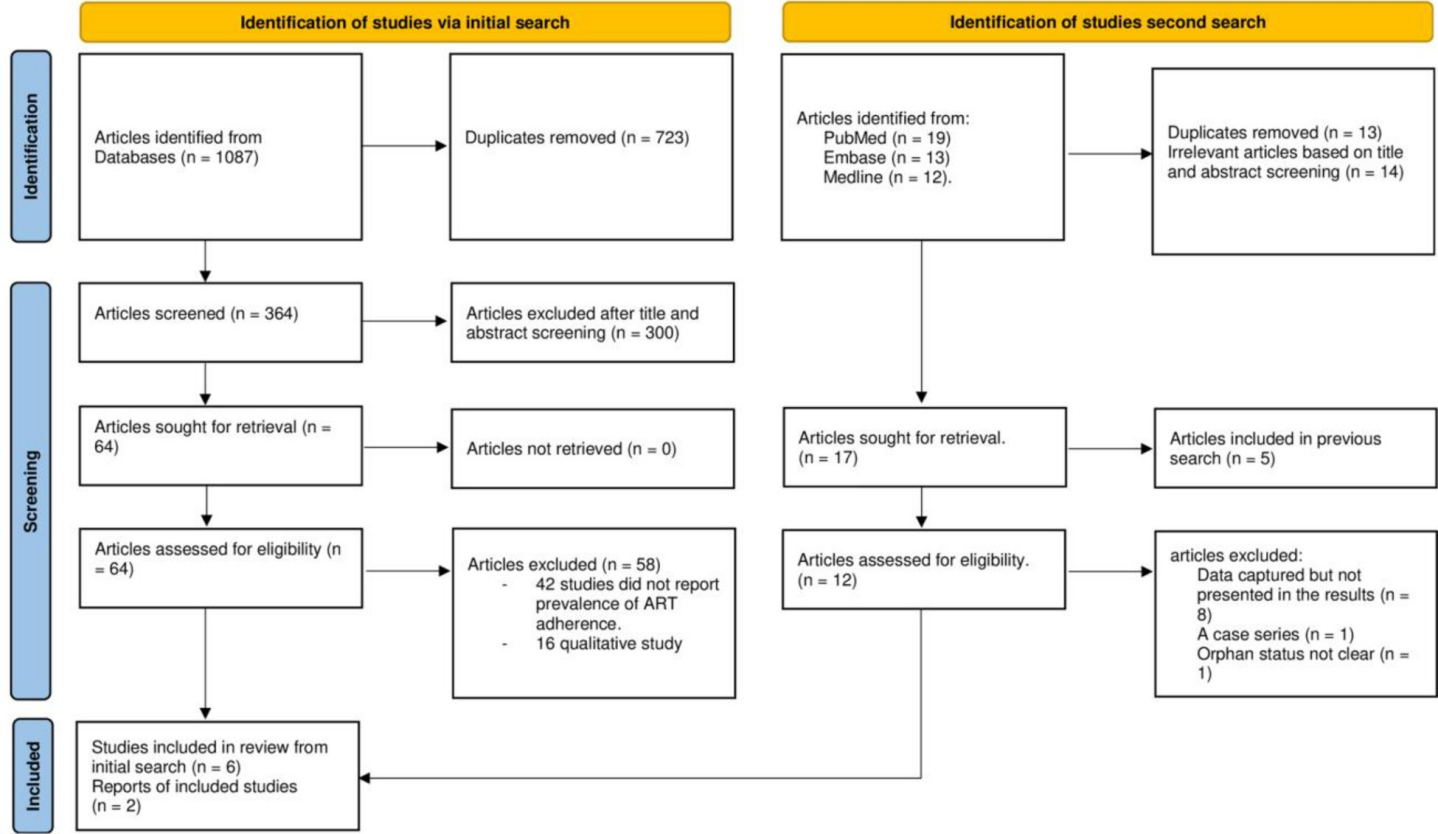
The PRISMA flow chart.

We retrieved 1087 articles from several databases during the initial data search, and 364 were duplicates. We eliminated 300 articles after screening “Titles and Abstracts”. We assessed the remaining 64 full articles for eligibility. A Second search was made in October 2023, and two articles were included. In the end, eight studies adhered to the study eligibility criteria **Fig 1**.

### Risk of bias assessment of the included papers

We used the nine-item JBI checklist for prevalence studies to evaluate the risk of bias and the quality of the included papers [20]. Note, baseline information from the cohort studies were included and thus, the JBI checklist for prevalence studies was used. Papers were assigned one point for each ‘*yes*’ response, and the rest were assigned zero points. The total score ranged from 0 to 9; a higher score represents good quality papers (**S1 Table)**. All authors confirmed the scores for each study.

### Data extraction

We designed a data extraction tool using Google Forms to capture and extracted information from the included studies. Extracted information included: (i) first author, (ii) publication year, (iii) year of data collection, (iv) country, (v) sample size, (vi) number of orphans, (vii) prevalence of adherence to ART, (viii) age of the participants, (ix) method for assessing adherence, (x) study design, and (xi) factors associated with adherence. SGK and JMS extracted the data independently; extracted data was cleaned by MMK, who also resolved any disparities in the data extraction process.

### Data analysis

Microsoft Office 2016 (Microsoft Inc., Washington, USA) and STATA 17.0 software (Stata Corp LLC, College Station, Texas, USA) were used for data cleaning and statistical analysis. Descriptive statistics was used to present individual study and participant characteristics. A random-effect model meta-analysis was used to determine the pooled prevalence of adherence to ART among children and adolescents based on the highest prevalence recorded from the various methods. The largest prevalence was chosen because only one study used a reliable method (NVP levels). The Higgins Inconsistency index (*I*^2^) was used to evaluate for heterogeneity among the selected studies, and the Galbraith plot was used to determine the number of studies responsible for the heterogeneity. In addition, the regression-based Egger test (determined the effect of small studies and publication bias), the nonparametric trim-and-fill method (determined the number of missing studies due to publication bias), and the Jackknife method (assess the effect of each study on the overall pooled prevalence using a leave one out option). Finally, meta-regression was used for continuous variables (i.e., sample size and JBI scores) and subgroup analysis for categorical variables (i.e., study design, continents, and country’s income status) to determine sources of heterogeneity. We performed additional random-effect models for the different prevalence reported by the ART adherence assessment methods if at least two studies reported the prevalence.

## Results

### Description of the included studies

A total of Eight papers (four cross-sectional and four cohort studies met the criteria for inclusion in this review. The studies recruited a total of 1458 orphans living with HIV out of 4204 children and adolescents sampled from five countries (Tanzania [n = 2] [12, 21], India [n = 2] [22, 23], Rwanda [n = 1] [13], Uganda [n = 1] [21], and Kenya [n = 3] [21, 24, 25]). Only two studies were outside the African continent [22, 23]. Based on World Bank Country income classifications, two studies involved patients from a low-income country–Rwanda [13] and Uganda [21], and the rest were from lower-middle-income countries (India, Nigeria, Kenya, and Tanzania) [12, 21–25]. The identified papers were published between 2006 [25] and 2019 [12]. However, the data was collected between 2001 [25] and 2015 [12] (**Table 1**).

**Table 1:** Study characteristics and prevalence of adherence among children and adolescent orphans.

Article	Study design (number of orphans)	Country (year of data collection)	Age of participants, mean (sd) in years	Adherence						Comment
				self-report (from caregivers or children)	Clinical attendance	NVP plasma levels	Pill count	Combined prevalence given	Highest reported prevalence	
Mugusi et al., 2019 [12]	CS (216)	Tanzania (2015)	9∙3 (3∙3)	79∙6%	82∙9%	72∙2%. Among these, 77∙6% were single orphans, and 22∙4% were double orphans			82∙9%	There was a significant relationship between NVP levels (cutoff of 3 μg/mL) and clinical attendance.
Bhattacharya et al. 2010 [22]	CS (73)	India (2008)	Range: 11–60 months	>95%	>90%		*	>95%	>95%	There was no statistically significant difference in ART adherence among the participants, orphans and non-orphan children and adolescents
Kikuchi et al. 2012 [13]	CS (371)	Rwanda (2011)	Range = 0∙5–14				50∙7% Double = 40∙7% Paternal = 53∙3% Maternal = 55∙1%		50∙7%	Orphaned children and adolescents, especially double orphans, were at a high risk of ART non-adherence, more so those with a sibling and non-biological individual as caretakers, due to lack of motivation.
Yoder et al. 2012 [24]	Retrospective Cohort (566)	Kenya (2007–2008)	Below 14		69∙3% in period 2 and 61∙5% in period 3		80∙9 in period 2 and 79∙1% in period 3		80∙9% in period 2 and 79∙1% in period 3.	Orphans had decreased ART adherence following election-related violence -period 2, and it decreased more with period 3 (four months to 1 year after the election crisis) Sample size varied across periods i.e., period 2 had fewer participants than period 3 and the sample varied across the different methods for measuring adherence. Highest sample size was considered in this review.
Bhattacharya et al., 2012 [23]	Cohort (40)	India (2006–2007)	Mean age = 7∙7 (2∙5)	*	*		*	85%	85%	There was no difference in ART adherence among the orphans and non-orphaned children. This was attributed to the role of extended family in India in caring for orphans.
Nyandiko et al. 2006 [25]	Cohort (106)	Kenya (2001–2005)	Range: 0∙4–13.7	*			*	73%	73%	There was no significant difference in ART adherence among the orphaned and non-orphaned children.
Akahara et al., 2017 [26]	CS (86)	Nigeria (2013)	Range: 10 months—15 years)	86.05% (93.75% among single orphans 63.64% samong double orphans)					86.05%	There was a significant difference in ART adherence among orphaned and non-orphaned children, with the double orphans having the lowest adherence level.
Vreeman et al., 2008 [21]	Cohort (1962)	Uganda, Kenya, and Tanzania (2010)	Range: <1 to 13 years							Odds for ART adherence was 0.919 (85% CI 0.864–0.979) orphan children compared to non-orphans

CS, Cross-sectional Study

### Methods used to assess adherence to ARVs by children and adolescent orphans living with HIV

The methods to assess for adherence were the use of pill counts (n = 5) [13, 22–25] and caregivers’ self-report (n = 5) [12, 21–23, 25], followed by clinical attendance (n = 4) [12, 22–24]. The least used method was NVP plasma level (n = 1) [12].

### Prevalence of adherence to ARVs by children and adolescent orphans living with HIV

Most studies (n = 3) provided the combined prevalence based on the different methods used to assess adherence [22, 23, 25]. The combined prevalence ranged between 73% [25] and >95% [22].

### The pooled prevalence of adherence to ART among children and adolescents

**Fig 2:**
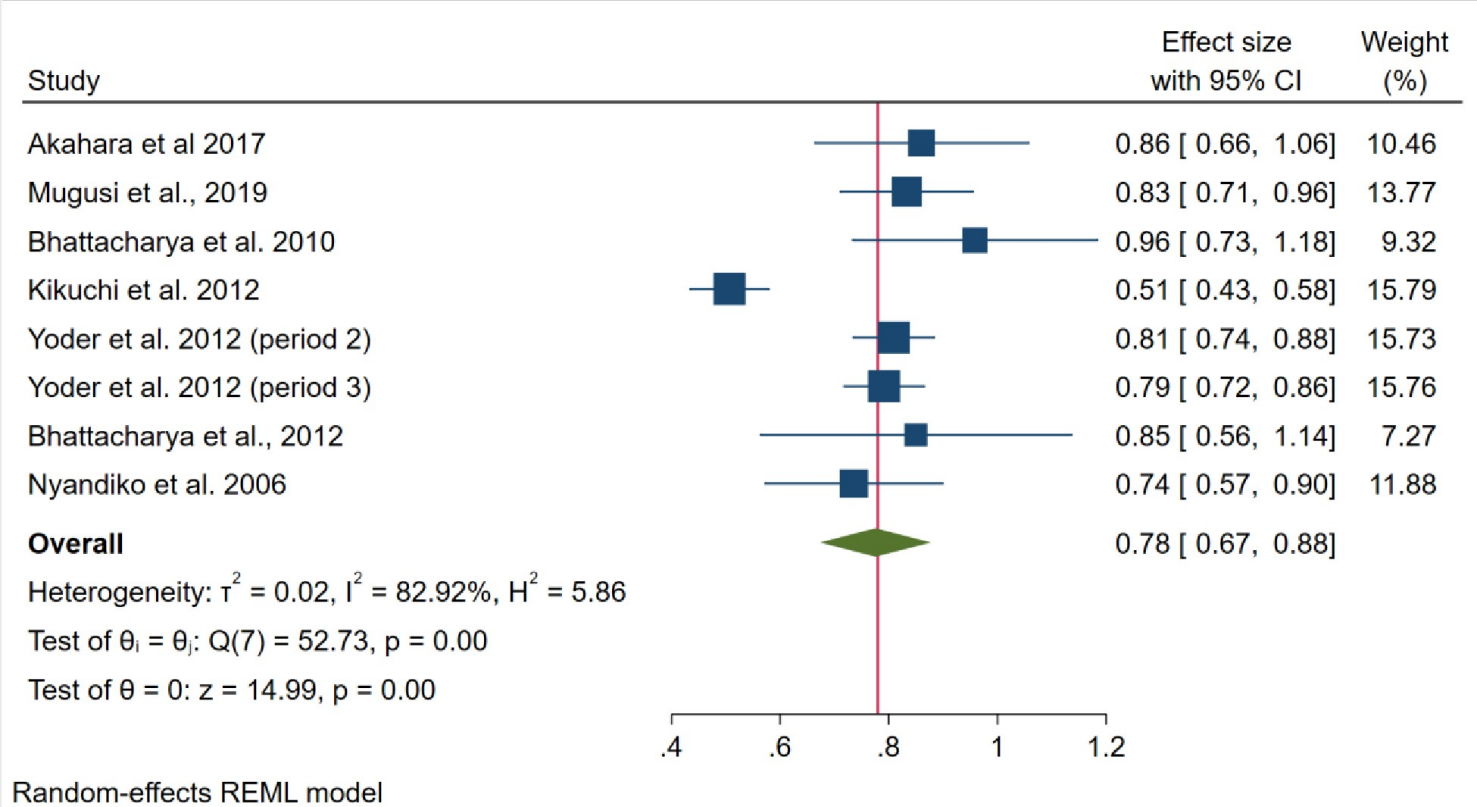
Forest plot showing the pooled prevalence of adherence to ART among orphan adolescents and children.

**Fig 3:**
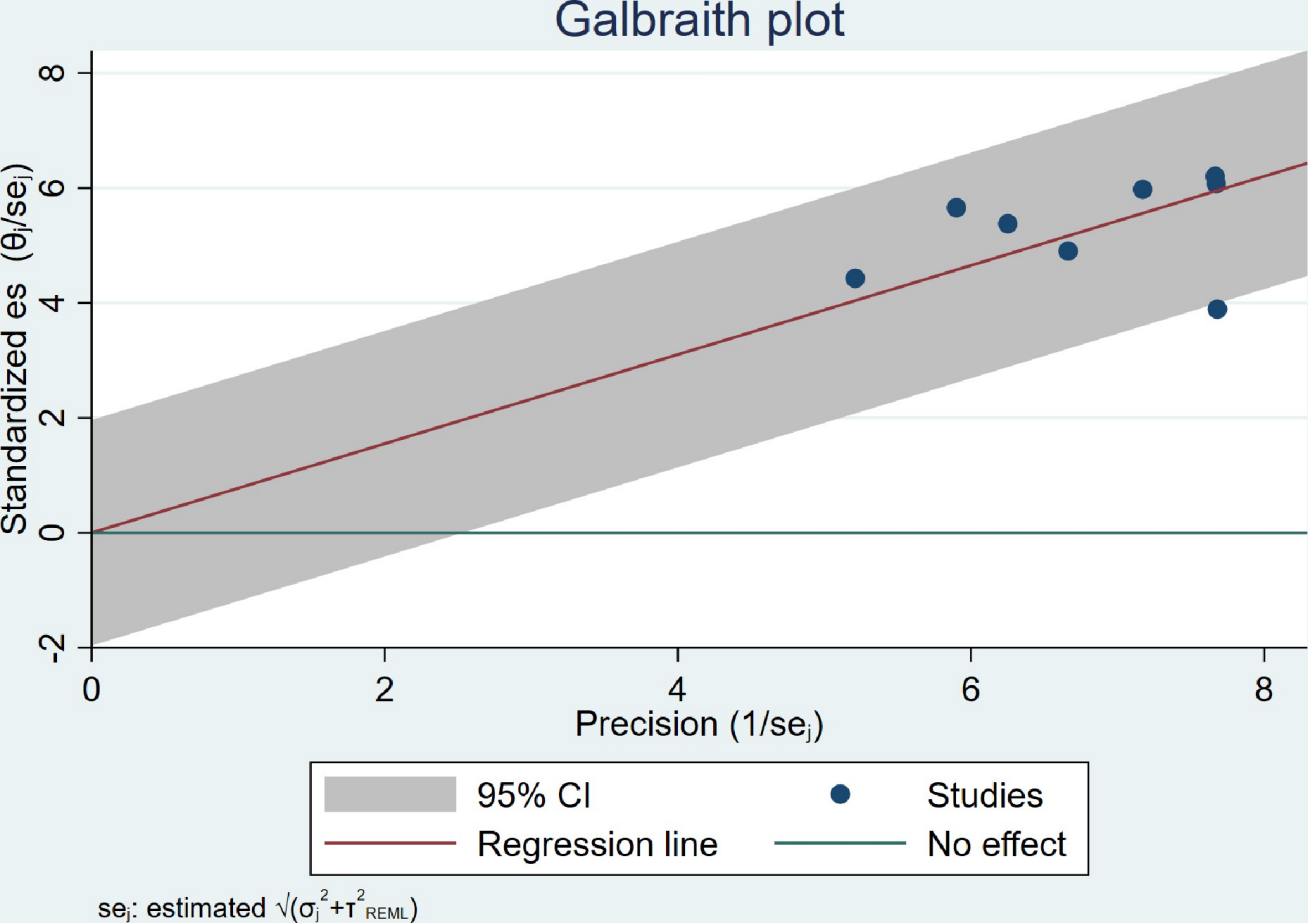
Galbraith plot for studies about ART adherence among orphan adolescents and children.

Based on the highest prevalence reported in the different studies, the pooled prevalence of adherence was 78.0% (95% CI: 67.4–87.7; I^2^ = 82.92%, *p*<0.001) (**Fig 2)** and based on Galbraith plot (**Fig 3**) one studies was an outlier. Despite the heterogeneity, there was no publication bias: the slope from egger’s test was 2.03 (standard error = 1.399), and the p-value was 0.1475. However, two studies were missing based on the Trim and Fill bias analysis. Based on the leave-one-out analysis, there was no evidence that a study substantially influenced the final pooled estimate. Both sample size and total score on JBI were not responsible for the heterogeneity based on meta-regression.

At subgroup analysis both country (Q beta = 51.73, *p*<0.001) and country income status (Q beta = 49.53, *p*<0.001) were responsible for the heterogeneity. The pooled prevalence was 81% in the LMICs and 51% in the LIC. Also, the pooled prevalence’s were 92%, 83%, 79%, and 51% in India, Tanzania, Kenya, and Rwanda, respectively. The pooled prevalence in Africa was 75%.

### Pill count

Two studies had prevalence based on this method of assessing adherence [13, 24]. The prevalence’s were 50.7% [13], 80.9% in period 2 [24], and 79.1% in period 3 [24]. However, other studies used the method.[22, 23, 25] Based on orphan status, paternal orphans had an ART adherence of 50.3%, while maternal orphans had an adherence of 53.3% [13]. The pooled prevalence 70.2% (95% CI: 51.0–89.5; I^2^ = 95.1%, *p*<0.001).

### Clinical attendance

Four studies had prevalence based on this method of assessing adherence [12, 22–24]. The prevalence’s were 82.9% [12], 70∙1% in period 2 [24], 61.5% in period 3 [24], and above 90% in a study done in India [22]. The pooled prevalence 81.3% (95% CI: 76.7–85.9; I^2^ = 0%, *p* = 0.711).

### Caregivers’ self-report

One study gave the prevalence based on this method of assessing adherence [12]. The prevalence was 79.6% [12]. However, the method was used by the other three studies [22, 23, 25]. The study by Nyandiko et al. (2006) captured either the caregiver or the patient reports [25].

### NVP plasma concentration

One study gave prevalence based on this method of assessing adherence.[12] The prevalence was 72.2% [12].

### Factors associated with adherence to ARVs by children and adolescent orphans living with HIV

Two studies studied factors associated with ART non-adherence among orphaned children and adolescents, reporting orphan status as a major contributor to non-adherence [12, 13]. A double orphan status was more likely to be associated with ART non-adherence, while the other orphanhood statuses were less likely to be associated with ART non-adherence [13]. However, for one study, [12] none of the other patients’ and caregivers’ social demographics and clinical characteristics were statistically significant in regression analysis. The other identified factors included: pill burden, caregiver involvement, stunting, and caregiver relationship [13]. Pill burden (≥3), low caregivers’ involvement, and less than standard deviation stunting were associated with ART non-adherence [13]. Children and adolescents with a grandparent or aunt/uncle as caregivers were more likely to have better ART adherence than children with a sibling as a caregiver [13].

### Relationship between the different orphan statuses and adherence

Compared to non-orphaned children, three studies found no statistically significant difference in ART adherence among the orphans [22, 23, 25]. However, four studies reported that orphaned children and adolescents were more likely to have ART non-adherence [12, 13, 21, 24].

## Discussion

The aim of this systematic review and meta-analysis was to examine the level of orphanhood on adherence to antiretroviral therapy (ART) among children and adolescents with HIV/AIDS. The included studies demonstrated a wide range of ART adherence prevalence, varying from 50.7% to >95% based on different assessment methods. The pooled prevalence for adherence among orphan children and adolescents was 78%, which falls significantly short of the UNAIDS target of 95% by 2025 [10, 27]. This finding highlights the urgent need for a systematic approach to improve care for orphans and reduce the stigma surrounding HIV/AIDS among both caregivers and the community.

The prevalence range observed in this review aligns with a previous systematic review focusing on ART adherence among individuals below 18 years of age (ranging from 49% to 100%) [28]. The similarities in prevalence may be attributed to the reliance on caregivers to assist children and adolescents with adherence to ART, as well as the inclusion of studies mostly comes from low- and middle-income countries (LMICs) where HIV/AIDS is prevalent. However, the prevalence range in the present study was narrower than that among children and adolescents from high-income countries (ranging from 20% to 100%) [29], but wider than that among individuals aged 50 years and older (ranging from 80% to 100%) [30]. These findings emphasize the crucial role of caregiver support in adolescents’ and children’s adherence to ART, as they often rely on caregivers for encouragement and assistance. Additionally, orphan adolescents face unique challenges during their rapid psychosocial and physical transition, which may further hinder their adherence to ART [3]. Therefore, addressing the serious adherence challenges among orphan children and adolescents requires increased resources and support.

Despite limited information on the factors associated with ART non-adherence among orphans, it is evident that adherence to ART is significantly lower among orphans compared to non-orphans [12, 13, 24]. Orphaned children also face additional challenges, including poor nutritional status, higher rates of severe immunosuppression, and increased prevalence of opportunistic infections when compared to their non-orphaned counterparts [3, 22, 23]. Furthermore, orphans are more likely to present late to hospitals for ART enrollment, highlighting the long-term impact of orphanhood on ART adherence among children and adolescents. To address these challenges, healthcare and public health providers must actively engage with caregivers of orphaned children to improve ART adherence. Low caregiver involvement, possibly due to a lack of understanding about the importance of adherence, has been identified as a significant barrier [13]. Sensitization efforts aimed at caregivers, emphasizing the need for ART uptake and providing adequate social support, can help overcome some of these barriers. Additionally, having a sibling as a caregiver has been identified as a significant factor contributing to ART non-adherence among orphans [13]. Therefore, healthcare providers should pay special attention to orphans living with HIV/AIDS and under the care of their siblings, implementing strategies to enhance ART uptake and adherence in this specific population.

The studies included in this review are predominantly from LMICs where the extended family structure is prevalent, which is believed to be more supportive to orphaned children [13, 31, 32]. However, it is important to note that cultural changes and specific circumstances, such as insurgency in Kenya, can impact the prevalence of ART adherence reported in a study [24]. It is likely that findings related to social structures, such as family types, orphanages, or foster systems, may vary in countries with different contexts. Therefore, the findings of this review are more applicable to LMICs with similar settings, and further investigations are needed to understand the burden of ART adherence among orphans in high-income countries.

Promoting high ART adherence in children and adolescent orphans is challenging due to factors related to their developmental immaturity, lack of knowledge about their disease, and specific eating habits [13]. Furthermore, their adherence is heavily reliant on caregivers [13, 14], whereas this dependency poses particular challenges for double orphans who do not have their parents as caregivers, resulting in higher levels of non-adherence [13]. Successful provision of HIV care requires a comprehensive test and treatment cascade that addresses potential drop-off at various stages and ensures linkage for re-engagement to support sustained adherence and retention. While this analysis focuses on the final stage of the cascade among orphans, it sheds light on key service gaps that need to be addressed to better support orphans living with HIV/AIDS. The significant discrepancy in reported adherence among orphans across different countries calls for urgent action, especially in regions such as East Africa, which has a high number of orphans living with HIV/AIDS.

### Limitations

There are several limitations to consider when interpreting the findings of this systematic review and meta-analysis. First, most of the included studies relied on self-reports for assessing ART adherence, which are subject to recall bias and may not provide accurate information. Secondly, the number of studies included in this review was limited, and they were conducted in specific regions of the world. Therefore, the findings may not fully represent the adherence patterns among orphans in other parts of the world. While the included studies employed various methods to measure adherence, it is important to note that caregiver reports and clinical attendance may overestimate the true level of adherence [33]. Interestingly, the prevalence rate based on NVP plasma levels was lower compared to other adherence measurement methods among orphan children and adolescents living with HIV/AIDS. These findings raise questions about the efficacy, specificity, and sensitivity of NVP plasma levels as a measure of adherence, beyond its use in medication pharmacokinetics. The choice of the most reliable method for assessing adherence to achieve the 2025 UNAIDS target remains unresolved. However, future research should aim to address these limitations and provide a more comprehensive understanding of ART adherence among orphan children and adolescents. Lastly, the study had high level of heterogeneity, a challenge with many prevalence systematic reviews and meta-analysis due to the various factors such as time, location, absence of randomisation/balancing out of cofounders, and other methodological concerns that introduce significant variations [34].

## Conclusion

Approximately four-fifths of orphan children and adolescents living with HIV/AIDS adhere to antiretroviral therapy (ART). However, this level of adherence falls significantly short of the UNAIDS target of 95% by 2025, highlighting the urgent need to improve ART adherence among this vulnerable population. To address this challenge and promote better health outcomes, several key recommendations are proposed:

- Strengthen caregiver support: Enhance caregiver training programs and establish support networks to empower caregivers in providing comprehensive support and guidance for orphan children and adolescents in adhering to their ART regimen.
- Increase education and awareness: Launch community-based education initiatives to raise awareness about the importance of consistent ART adherence, combat stigma surrounding HIV/AIDS, and promote a supportive environment for orphaned individuals.
- Tailor interventions for double orphans: Develop targeted interventions specifically designed to address the unique challenges faced by double orphaned children and adolescents, considering their increased vulnerability and potential psychosocial stressors.
- Implement comprehensive care models: Establish integrated healthcare models that go beyond medication provision, encompassing psychosocial support, mental health services, nutritional assistance, and educational support, to address the multifaceted needs of orphaned children and adolescents.
- Enhance mental health and psychosocial support: Prioritize the provision of mental health services and psychosocial support programs to address the emotional well-being and resilience of orphaned individuals, recognizing the impact of psychosocial factors on ART adherence.
- Improve access to healthcare services: Take measures to enhance access to healthcare services by expanding the reach of ART clinics, ensuring reliable medication supply chains, and implementing strategies to minimize barriers to care, such as transportation or financial constraints.
- Foster collaboration and partnerships: Encourage collaborations between healthcare providers, community organizations, governmental agencies, and non-governmental organizations to leverage resources, share best practices, and implement comprehensive strategies aimed at improving ART adherence among orphaned children and adolescents.

By implementing these recommendations, we can pave the way for improved ART adherence, better health outcomes, and enhanced overall well-being for orphan children and adolescents living with HIV/AIDS. This concerted effort will contribute to reducing HIV transmission rates, achieving the UNAIDS targets, and ensuring that every child and adolescent affected by HIV/AIDS receives the care and support they need to thrive.

## Supporting information

S1 Checklist (DOCX)

S1 Table Risk of bias and quality assessment for included studies. (DOCX)

S1 File PubMed search string. (DOCX)
