# Supplementary material for: The level of antiretroviral therapy (ART) adherence among orphan children and adolescents living with HIV/AIDS: A systematic review and meta-analysis
Source: PLoS One. 2024 Feb 21;19(2):e0295227. doi: 10.1371/journal.pone.0295227 (PMC10881004; doi:10.1371/journal.pone.0295227)
Supplement: S1 Table — (DOCX) [file pone.0295227.s002.docx]

(((((Adherence)) OR (Compliance[MeSH Terms])) AND ((HIV[MeSH Terms]) OR (AIDS[MeSH Terms]))) AND (orphan*)) AND (((((((((pediatrics) OR (paediatrics)) OR (child*)) OR (adolescent*)) OR (teenager*)) OR (juvenile)) OR (minors)) OR (emancipated)) OR (infant))
