# Supplementary material for: The level of antiretroviral therapy (ART) adherence among orphan children and adolescents living with HIV/AIDS: A systematic review and meta-analysis
Source: PLoS One. 2024 Feb 21;19(2):e0295227. doi: 10.1371/journal.pone.0295227 (PMC10881004; doi:10.1371/journal.pone.0295227)
Supplement: S1 File — (DOCX) [file pone.0295227.s003.docx]

| Author and year | Was the sample frame appropriate to address the target population? | Were study participants sampled in an appropriate way? | Was the sample size adequate? | Were the study subjects and the setting described in detail? | Was the data analysis conducted with sufficient coverage of the identified sample? | Were valid methods used for the identification of the condition? | Was the condition measured in a standard, reliable way for all participants? | Was there appropriate statistical analysis? | Was the response rate adequate, and if not, was the low response rate managed appropriately? | Total score | Comment/s |
| --- | --- | --- | --- | --- | --- | --- | --- | --- | --- | --- | --- |
| Mugusi et al. 2019  ^1^ | Yes | No | Yes | Yes | Yes | Yes | Yes | Yes | Yes | 8 |  |
| Bhattacharya et al. 2010 ^2^ | Yes | Yes | Yes | Yes | Yes | Yes | Yes | Yes | Yes | 9 | Despite using various methods to assess adherence. They did not specify the prevalence obtained from the different methods |
| Kikuchi et al. 2012 ^3^ | Yes | Yes | Yes | Yes | Yes | Yes | Yes | Yes | Yes | 9 |  |
| Akahara et al., 2017 ^4^ | Yes | NC | Yes | No | Yes | No | No | Yes | Yes | 5 |  |
| Yoder et al. 2012 ^5^ | Yes | Yes | Yes | Yes | Yes | Yes | Yes | Yes | Yes | 9 |  |
| Bhattacharya et al. 2012 ^6^ | No | Yes | Yes | Yes | Yes | Yes | Yes | Yes | Yes | 8 | Despite using various methods to assess adherence. They did not specify the prevalence obtained from the different methods |
| Nyandiko et al. 2006 ^7^ | Yes | Unclear | Yes | Yes | Yes | Yes | Yes | Yes | No | 7 | Despite using various methods to assess adherence. They did not specify the prevalence obtained from the different methods |

1. Mugusi SF, Mopei N, Minzi O. Adherence to combination antiretroviral therapy among orphaned children in Dar es Salaam, Tanzania. *South Afr J HIV Med* 2019; **20**(1): 954.

2. Bhattacharya M, Rajeshwari K, Saxena R. Demographic and clinical features of orphans and nonorphans at a pediatric HIV centre in North India. *Indian J Pediatr* 2010; **77**(6): 627-31.

3. Kikuchi K, Poudel KC, Muganda J, et al. High risk of ART non-adherence and delay of ART initiation among HIV positive double orphans in Kigali, Rwanda. *PLoS One* 2012; **7**(7): e41998.

4. Akahara C, Nwolisa E, Odinaka K, Okolo S. Assessment of Antiretroviral Treatment Adherence among Children Attending Care at a Tertiary Hospital in Southeastern Nigeria. *J Trop Med* 2017; **2017**: 3605850.

5. Yoder RB, Nyandiko WM, Vreeman RC, et al. Long-term impact of the Kenya postelection crisis on clinic attendance and medication adherence for HIV-infected children in western Kenya. *Journal of acquired immune deficiency syndromes (1999)* 2012; **59**(2): 199-206.

6. Bhattacharya M, Saxena R. Outcome of anti-retroviral treatment in HIV-infected orphans and non-orphans at an ART centre in North India. *Paediatr Int Child Health* 2012; **32**(4): 228-32.

7. Nyandiko WM, Ayaya S, Nabakwe E, et al. Outcomes of HIV-infected orphaned and non-orphaned children on antiretroviral therapy in western Kenya. *Journal of acquired immune deficiency syndromes (1999)* 2006; **43**(4): 418-25.

8. Vreeman RC, Ayaya SO, Musick BS, et al. Adherence to antiretroviral therapy in a clinical cohort of HIV-infected children in East Africa. *PLOS ONE* 2018; **13**(2): e0191848.
